# Parental Stress and Caregiver Role Modulate Child–Caregiver Prosodic Synchrony in Autism: A Computational Analysis

**DOI:** 10.1002/aur.70189

**Published:** 2026-01-27

**Authors:** Maria Grazia Logrieco, Giulio Bertamini, Laura Casula, Mohamed Chetouani, Silvia Guerrera, Mirco Fasolo, Paola Venuti, Maria Luisa Scattoni, Francesca Fulceri, Stefano Vicari, David Cohen, Giovanni Valeri

**Affiliations:** ^1^ Child and Adolescent Neuropsychiatric Unit Bambino Gesù Children's Hospital, IRCSS Rome Italy; ^2^ Department of Humanities University of Foggia Italy; ^3^ Department of Child and Adolescent Psychiatry Pitié‐Salpêtrière University Hospital, Sorbonne University Paris France; ^4^ Institute of Intelligent Systems and Robotics Sorbonne University Paris France; ^5^ Department of Psychology University G. d'Annunzio Chieti Italy; ^6^ Department of Psychology and Cognitive Science University of Trento Rovereto Italy; ^7^ Research Coordination and Promotion Service Istituto Superiore Di Sanità Rome Italy; ^8^ Life Sciences and Public Health Department Catholic University Rome Italy

**Keywords:** artificial intelligence, autism, caregiver role, interaction, parents, prosodic synchrony, stress

## Abstract

Parental stress influences parent–child interactions in typical development and is a prognostic factor of autism outcome. However, we still do not know to what extent parental stress affects parent–child interactions and whether caregiver role matters. This study explored the relationship between parental stress and prosodic synchrony in parent–child vocal interactions, drawing on complex dynamic systems and affective computing frameworks. We assessed 62 dyads (31 autistic preschoolers, interacting separately with their mother and father) during structured play interactions at two time points (12 months apart) along with perceived parental stress. We used a Deep Learning model to segment child‐caregiver acoustic interactions with high accuracy automatically. Downstream, prosodic synchrony was modeled through cross‐recurrence quantification analysis. Linear mixed‐effects models were used to assess the impact of parental stress, caregiver role, and time on synchrony metrics. Models showed significant associations between parental stress and synchrony metrics for spectral and formant amplitude features. Higher stress levels were linked to less stable, predictable, and structured interactions. These effects were more pronounced in father–child dyads compared to mother–child dyads. Permutation analyses confirmed that these associations were specific to moment‐to‐moment coordination rather than general acoustic similarity. In autistic children, parental stress levels are linked with the temporal dynamics of parent–child prosodic synchrony, specifically affective speech and moment‐to‐moment coordination. It appears to be more pronounced in fathers. The results underscore the importance of fostering parental well‐being and tailoring interventions to account for differences between maternal and paternal interaction patterns in autism.

## Lay Summary

Parents of autistic children often experience high levels of stress, which can affect how they communicate with their child.

This study found that when parents—especially fathers—feel more stressed, their communication with their child becomes less coordinated and more difficult to follow.

Supporting parents' mental health could help improve how they connect and communicate with their autistic children.

The study employed artificial intelligence techniques to analyze parent–child vocal interactions.

## 1 Introduction

### 1.1 Synchrony in Child–Caregiver Interactions

The quality of child–caregiver interactions is a cornerstone of developmental outcomes across the lifespan, underscoring the foundational role of relational experiences in early development (Guralnick 1999; Humphreys et al. 2024; Karmiloff‐Smith 2010; Karmiloff‐Smith et al. 2012). This quality is largely determined by the degree of attunement, responsiveness, and synchrony within the dyadic relationship (Leclère et al. 2014; Shonkoff et al. 2000). Attunement refers to the caregiver's ability to perceive and interpret the child's internal states accurately. Responsiveness reflects timely and appropriate behavioral reactions to the child's cues. Interpersonal synchrony, defined as the dynamic and reciprocal coordination of behavior, affect, and physiological rhythms between individuals, is a fundamental process that facilitates bonding, co‐regulation, and shared intentionality (Feldman 2012a), indicating mutual adaptation within the interaction. Co‐regulation refers to how caregivers and children work together to manage emotions, behaviors, and attention in real time, with the caregiver scaffolding the child's regulation while adapting to the child's needs (Davis et al. 2018). Synchrony occurs on multiple levels, behavioral, physiological, and neural, and reflects moment‐to‐moment mutual coordination in the dyad (Feldman 2012a, 2012b). Synchrony dynamics lay the groundwork for self‐regulated behavior, empathy, and social competence (Davis et al. 2018; DePasquale 2020; MacPhee et al. 2015) during child development. Synchrony has initially been studied using linear approaches, such as cross‐correlation, but accumulating evidence indicates that interpersonal exchanges are dynamically complex and require methods capable of capturing non‐linear, time‐lagged, and asymmetric patterns. Mathematically, synchrony has therefore been explored using non‐linear dynamic systems theory and time‐series analysis, which reveal how cycles of engagement and disengagement are temporally patterned (Delaherche et al. 2012). Importantly, research indicates that the relationship between synchrony and developmental outcomes is also non‐linear: more synchrony is not always better. Optimal synchrony lies within a functional range: too little may signal disengagement or misattunement, whereas too much may be intrusive or lead to enmeshment, potentially disrupting affect regulation (Bowsher‐Murray et al. 2023; Dahan et al. 2016; Galbusera et al. 2019; Mayo and Gordon 2020).

This dynamic and reciprocal interpersonal coordination is present in both typically developing (TD) children and in children with neurodevelopmental disorders although the way it manifests and how effectively it supports development may vary. In TD children, interpersonal synchrony tends to follow predictable and reciprocal patterns, allowing developmental processes to unfold in a fluid, responsive context (Feldman 2012a, 2012b). In contrast, child–caregiver interactions in autism can be more variable and effortful (Kellerman et al. 2020), even before a formal diagnosis has been given (Saint‐Georges et al. 2011). Autism is a complex neurodevelopmental condition characterized by early difficulties in social reciprocity, communication, and restricted, repetitive patterns of behavior (APA 2022; Lord et al. 2020). In autistic children, synchrony is frequently altered (Cohen et al. 2013; McNaughton and Redcay 2020; Pelphrey et al. 2011). These atypical patterns may interfere with experience‐dependent learning mechanisms that are essential for acquiring social, communicative, and cognitive skills (Kuo et al. 2023; Nelson et al. 2024). For example, some research suggested that mothers of infants at increased likelihood of being autistic compensate for their child's reduced engagement by amplifying their efforts to maintain synchrony, using exaggerated gestures or vocalizations to capture the child's attention (Cohen et al. 2013; Steiner et al. 2018; Talbott et al. 2016). Crucially, levels of interpersonal synchrony during early interactions have been associated with later language development and socio‐emotional competencies, highlighting its predictive power across developmental trajectories (Kellerman et al. 2020; Siller and Sigman 2002, 2008). Importantly, the quality and dynamics of caregiver‐child synchrony are shaped by both child characteristics and caregiver factors, including parental well‐being and stress levels, which remain relatively understudied in relation to their impact on parent–child interaction dynamics.

### 1.2 Parental Stress, Roles, and Child–Caregiver Interaction in Autism

Building on the role of child–caregiver synchrony, it is therefore critical to examine how variables such as parenting stress may influence the interpersonal exchange. Parenting stress is defined as an “aversive psychological reaction to the demands of being a parent” (Deater‐Deckard 1998, 315), often arising when the perceived demands of caregiving exceed the available personal and social resources (Abidin 2012). Parents of autistic children consistently report higher levels of stress (Hayes and Watson 2013). This elevated stress frequently emerges before diagnosis, triggered by early concerns about the child's social or communicative development (Reed and Osborne 2012). This heightened stress can affect both the child and the parent, and is associated with poorer treatment outcomes (Osborne et al. 2008), increased behavioral challenges (Lecavalier et al. 2006), parental mental health issues (Hastings 2003), and less effective implementation and efficacy of interventions (Osborne and Reed 2010; Stadnick et al. 2015; Watson et al. 2017). Stress affects not only individual well‐being but also the quality of parent–child interactions. Elevated stress is linked to decreased sensitivity, increased intrusiveness, and lower patience in caregiving (Dolev et al. 2009; Zaidman‐Zait et al. 2014). Over time, these reciprocal influences can solidify and mutually reinforce altered interaction patterns (Fogel 2009; Sameroff 2009). Specific to synchrony, research investigated the impact of parental stress on child–caregiver synchrony primarily focusing on neural and physiological measures. These studies suggest that higher parental stress levels may alter the alignment of affective, cognitive, and physiological processes between caregiver and child. Such disruptions in interpersonal synchrony may, in turn, hinder children's capacity for emotional regulation and reduce their ability to engage in adaptive social interactions (Liu et al. 2022; Azhari et al. 2019, 2022; Im‐Bolter et al. 2015). In contrast, behavioral synchrony remains relatively underexplored, leaving open questions about how stress influences observable moment‐to‐moment interactions.

Moreover, the quality of caregiver‐child exchanges is associated with attachment processes, which provide a foundation for the child's emerging social, emotional, and cognitive skills (Cossette‐Côté et al. 2022).

Given the strong influence of parenting stress on interaction quality, interventions that support caregivers' emotional and behavioral regulation may help restore adaptive parent–child exchanges and enhance developmental outcomes (Hajal and Paley 2020; McDowell and Parke 2009). Engaging parents as partners in their child's learning process can also improve the generalization and maintenance of skills as well as family outcomes (Schreibman et al. 2015; Stahmer and Pellecchia 2015).

Parenting research has traditionally centered on maternal interactional behaviors such as responsiveness and structuring, which have been consistently linked to positive child outcomes. However, emerging evidence underscores the importance of including fathers, who bring a complementary set of interactional strengths to the caregiving relationship (Bentenuto et al. 2020). Despite their critical role in child development, fathers remain markedly underrepresented in intervention research (Flippin and Crais 2011; Fox et al. 2015; Oppenheim et al. 2025; Rankin et al. 2019).

From an interactional perspective, fathers tend to engage in more horizontal interactions that are reciprocal and play‐based. These behaviors complement maternal styles, which are often oriented toward more vertical interactions aimed at guidance and didactic exchanges (Feldman 2003; Paquette 2004). Intervention research has shown that both parents can improve their structuring and non‐intrusiveness abilities, but fathers often exhibit greater gains in promoting child involvement in autonomy, initiative, and peer‐like engagement (Bentenuto et al. 2020; Siller and Sigman 2008).

### 1.3 Computational Methods and Speech Processing

Behavioral analysis traditionally relies on observational methods, especially in developmental contexts where non‐invasiveness is of primary concern. Recently, the analysis of child–caregiver synchrony has benefited from computational methods, enabling fine‐grained analysis of temporal coordination and interactional dynamics (Bourvis et al. 2018; Delaherche et al. 2012). However, observational approaches are time‐consuming and labor‐intensive, which limits their scalability and translational potential. In the computational domain, affective computing and speech processing were employed to understand parent‐infant dynamics and developmental processes, with a focus on prosodic features and affective content (Oller et al. 2013; Weisman et al. 2015). Speech cues have also been shown to be relevant in autism for diagnostic (Eni et al. 2025; Godel et al. 2023) and developmental aspects (Cohen et al. 2013; Lahiri et al. 2022; Warlaumont et al. 2014), as well as in the clinical interaction (Bone et al. 2014).

Methods grounded in complex systems theory provide robust tools for quantifying interpersonal coordination from a non‐linear perspective. Cross‐recurrence quantification analysis (CRQA) is one such method, assessing how two systems dynamically coordinate over time by identifying recurring patterns in their joint activity. Unlike linear techniques, CRQA captures complex, time‐lagged, and asymmetric forms of coupling, providing insights into the structure, stability, and variability of interactions. Its robustness to noise and non‐stationarity makes it particularly suited for naturalistic dyadic data, such as caregiver‐child vocal exchanges. CRQA has been successfully applied to model temporal alignment in behavioral interactions (Coco and Dale 2014; Shockley 2005), conversational synchrony (Fusaroli and Tylén 2016), emotional contagion across multimodal channels (Varni et al. 2017), and child‐caregiver vocal interactions relevant for language development (Duong et al. 2024; Warlaumont et al. 2014). Key CRQA metrics quantify different aspects of coordination: how often partners align, the predictability of their interactions, the presence of sustained shared states, and the balance between stable and evolving coordination patterns. By capturing how interacting partners revisit similar states over time, CRQA provides a fine‐grained, data‐driven framework to quantify both the frequency and the temporal organization of interpersonal synchrony, revealing aspects of coordination not accessible through linear methods.

Integrating such methods into longitudinal clinical research remains uncommon, as their automated application has high temporal resolution. Further, few studies have examined how synchrony evolves as a function of caregiver variables such as stress and caregiver role.

### 1.4 Aim

The present study examines child–caregiver acoustic synchrony with a focus on prosodic and affective dimensions across a longitudinal cohort of autistic preschoolers. Specifically, we explore caregiver‐related differences, the temporal evolution of synchrony dynamics, and, critically, the influence of parental stress. We hypothesized that parental stress and caregiver role would shape child–caregiver interaction dynamics, influencing both the degree of patterned recurrences and the proportion of sustained shared states in vocal coordination in prosodic features. Building on these hypotheses, the study is guided by the following research questions: (1) whether child–caregiver acoustic synchrony patterns, specifically the balance between predictable sequences and overall recurrence, change over a 12‐month period in dyads with autistic preschool children; (2) whether the balance between sustained versus patterned recurrences in prosodic coordination differs as a function of caregiver role; (3) whether parental stress levels are associated with longitudinal variation in acoustic synchrony metrics, particularly in the balance between predictable versus sustained coordination patterns; and (4) the extent to which these aspects reflect moment‐to‐moment coordination versus synchronization operating at a more general prosodic level.

To address these questions, we optimized an AI‐driven system for second‐by‐second automated segmentation of naturalistic child–adult acoustic interactions and developed an analytic pipeline integrating complex dynamic systems with repeated‐measures linear mixed models (LMMs). To quantify acoustic synchrony, we extracted a set of prosodic features from both child and adult vocalizations to create continuous time series for each participant. These feature time series served as input to CRQA, allowing us to measure the temporal coordination of vocal patterns across child and caregiver. Given the novelty of applying these computational and CRQA‐based methods to child–caregiver prosodic interactions, the analyses are exploratory, focusing on a limited set of interpretable metrics to provide initial insights into the temporal organization of interpersonal coordination. In this study, we specifically focused on the balance between predictable sequences and overall recurrence and the balance between sustained versus patterned recurrences, prioritizing metrics that are directly interpretable while still providing a rich description of the temporal organization, stability, and flexibility of coordination between child and adult vocalizations without relying on a priori hypotheses.

To further strengthen the robustness of exploratory findings, we employed a permutation analysis to ensure that the observed patterns reflect genuine interpersonal alignment rather than random or spurious correlations. Finally, by integrating non‐linear CRQA synchrony metrics with longitudinal linear models, this study captures the complexity and temporal structure of interpersonal coordination while ensuring interpretability. This approach enables developmentally meaningful insights into how caregiver stress and caregiver role relate to changes in child–caregiver vocal coordination over time. To our knowledge, this is the first study to computationally explore the temporal interplay between prosodic and affective synchrony, parental stress, and caregiver factors in a longitudinal design leveraging high‐precision automated analysis.

## 2 Methods

### 2.1 Participants

Children were recruited at the Child and Adolescent Psychiatry Unit of Bambino Gesù Children's Hospital (OPBG) among the sample enrolled in a 24‐month longitudinal single‐blinded, two‐arm randomized parallel‐group aimed at comparing the efficacy of two different treatment approaches. Eligible participants were children aged below 36 months with an ASD diagnosis based on clinical judgment according to the Diagnostic and Statistical Manual of Mental Disorders Fifth Edition (DSM‐5), supported by the Autism Diagnostic Observation Schedule‐Second Edition (ADOS‐2; Lord et al. 2012) administered by clinicians trained to reliability. Exclusion criteria were: (1) diagnosis of epilepsy or history of seizures, (2) presence of known genetic syndromes associated with ASD, (3) presence of severe cardiovascular, organic, and systemic diseases, and medical conditions that may affect brain development or the infant's ability to participate in the study. The sample consists of 62 dyads (31 autistic preschoolers, each with their mother and father) who completed a structured experimental test designed to collect child–caregiver interaction audio signals (see *Experimental Testing* ).

The mean age of children at enrolment was 28.88 ± 5.85 months, and 26 were male (84%). The mean paternal age was 42.84 ± 11.14 years, and the mean maternal age was 40.81 ± 10.14 years. The parental sample consisted of four fathers and six mothers of Asian ethnic backgrounds, whereas the remaining parents were Italian. Parental job employment consisted of supervisory or technical positions (semi‐routine) (3 mothers), intermediate positions as small employers or accountants (12 mothers, 20 fathers), managerial/professional jobs (one mother, 12 fathers). Sixteen mothers were stay‐at‐home parents.

### 2.2 Clinical Testing

Autism Diagnostic Observation Schedule‐Second Edition (ADOS‐2, Lord et al. 2012), Griffith Mental Developmental Scale‐Extended Revised (GMDS‐ER, Luiz et al. 2006), Vineland Adaptive Behavior Scales‐Second Edition (VABS‐II, Chatham et al. 2018), and Parent Stress Index (PSI, Abidin et al. 2006) were administered by licensed clinical psychologists blinded to the play experimental test results. Clinical data were extracted from individual clinical records and summarized in Table 1. Data from the PSI were computed for both mother and father and included in the statistical analysis plan. PSI is a widely used standardized measure designed to evaluate stress levels experienced by parents related to their parenting role. In detail, the PSI assesses three primary domains of parenting stress: (1) parental distress, measuring personal stress levels experienced by the parent; (2) parent–child dysfunctional interaction, examining parental perceptions of difficulties in interactions with their child; and (3) difficult child, reflecting parental perceptions regarding the child's behavioral challenges. Scores across these three domains yield an overall Parenting Stress Index (PSI Total Score), providing a comprehensive evaluation of stress in the parenting role. Longitudinal test–retest reliability of the PSI across T1 and T2, assessed with a two‐way consistency intraclass correlation coefficient (ICC), was good for both mothers (ICC = 0.79; 95% CI = [0.58, 0.90], *p* < 0.001) and fathers (ICC = 0.73; 95% CI = [0.47, 0.87], *p* < 0.001).

**TABLE 1:** Clinical characteristics of the sample.

	Baseline		T1		T2	
	Mean	SD	Mean	SD	Mean	SD
PSI Total Score mother	81.87	19.68	79.22	25.31	80.50	22.14
PSI Total Score father	—	—	76.94	21.05	85.06	26.66
ADOS‐2 CSS	6.43	1.31	6.51	1.38	6.59	1.62
GMDS‐ER GDQ	69.16	13.49	68.88	14.92	72.56	19.30
VABS‐II—CS	60.11	13.50	55.22	16	52.94	14.37

Abbreviations: ADOS‐2, Autism Diagnostic Observation Schedule Second Edition; CS, composite score; CSS, calibrated severity scores; GDQ, general developmental quotient; GMDS, Griffith mental development scales; PSI, parenting stress index; T1, 12 months from starting treatment; T2, 24 months from starting treatment; VABS‐II, Vineland adaptive behavior scales II edition.

### 2.3 Experimental Testing

#### 2.3.1 Collection of Child–Caregiver Interaction Audio Signals During Free Play Sessions

Sessions occurred at time points established for convenience within the framework of the pre‐existing clinical trial (T1: 12 months after the intervention began; T2: 24 months after the intervention began). A licensed clinical psychologist, blinded to trial procedure and group assignments, performed an assessment of free play interactions between parents and children. The play session was structured following the “parent–child interaction” (Kasari et al. 2012) with a standardized set of toys. Parents were instructed to “play with their child as they play with them at home.” During the play session, parents interacted in a specific sequence: in the first phase, one parent actively plays with the child while the other observes; in the second phase, the parents switch roles. Each phase lasted 5 min. The experimenter instructed the parents when to interrupt the interaction and take over. Data for each child were deemed complete with available recordings of both parents at both assessment time points, resulting in a total of four recordings per child. Each session was conducted in a quiet hospital room and videorecorded.

#### 2.3.2 Automated Speech Segmentation

A deep learning (DL) system for two‐layer classification, trained on naturalistic clinical data, performed the automated segmentation of child–caregiver interaction audio signals. As validated by Bertamini et al. (2025), it consists of two siamese neural networks designed and trained to perform the second‐by‐second similarity‐based classification using Mel‐frequency Cepstral coefficient (MFCC) spectrogram features. The first layer detects human voice presence, while the second handles speaker diarization. The system was trained on noisy, unstructured clinical interactions between autistic preschoolers and clinicians and is able to process the non‐linguistic vocalizations typical of this population. It was validated through a robust cross‐validation procedure and showed optimal performance in voice activity detection under diverse conditions. Speaker diarization also performed well. It also proved reliable when compared to two additional raters under masked conditions. Fliess's *k* coefficient between the three sets of annotations was *k* = 0.87, reflecting strong agreement. Finally, the DL model could be rapidly adapted to new contexts and data with just a few annotated examples, demonstrating high scalability and flexibility.

In this study, ongoing reliability for speaker diarization was assessed by masked manual annotation of *N* = 150 randomly sampled 1‐s audio segments, balanced for speaker and session. Classification performance (true positives = 71, true negatives = 65, false positives = 2, false negatives = 13) indicated balanced accuracy = 0.91, precision = 0.83, sensitivity (recall) = 0.97, F1‐score = 0.90, specificity = 0.85, Matthews correlation coefficient (MCC) = 0.81, receiver operating characteristic area under the curve (ROC‐AUC) = 0.91, and precision‐recall area under the curve (PR‐AUC) = 0.82. Cohen's kappa (*k* = 0.80) indicated good inter‐rater reliability.

#### 2.3.3 Feature Extraction

Child–caregiver acoustic features were extracted using openSMILE (open‐source speech and music interpretation by large‐space extraction). It consists of a widely used framework for automatic extraction of several acoustic features from speech, music, and environmental audio, at different levels, including low‐level and high‐level descriptors (Eyben et al. 2010). OpenSMILE has been extensively used in research, particularly in the domains of speech analysis and affective computing, as well as in clinical research (Fusaroli et al. 2017; Oller et al. 2013; Schuller et al. 2013). We used the specific set derived for paralinguistic and affective speech analysis, including clinical aspects (eGeMAPSv0: Eyben et al. 2015). The 25 OpenSMILE low‐level descriptors employed in this study are summarized in Table 2.

**TABLE 2:** openSMILE low‐level descriptors from the eGeMAPSv02 feature set (Eyben et al. 2015).

Feature	Description
Loudness	Perceived intensity of sound, related to amplitude
alphaRatio	Ratio of energy in the low frequencies (alpha range)
hammarbergIndex	Spectral balance, indicating presence of harmonics in speech
slope0–500	Spectral slope in the 0–500 Hz range, relating to voice quality
slope500–1500	Spectral slope between 500 and 1500 Hz, indicative of vowel quality and harmonics
spectralFlux	Rate of change in the spectrum, related to sound texture and dynamics
mfcc1 to mfcc4	First four Mel‐Frequency Cepstral Coefficients (MFCCs)
F0semitoneFrom27.5 Hz	Fundamental frequency (F0) deviation from 27.5 Hz in semitones, indicating pitch change
jitterLocal	Local pitch variability (jitter), indicating roughness or instability in voice
shimmerLocaldB	Local amplitude variation (shimmer), indicating breathiness or roughness
HNRdBACF	Harmonics‐to‐noise ratio (HNR), reflecting vocal fold vibration smoothness
logRelF0‐H1‐H2	Logarithmic ratio between F0 and harmonic components, related to voice quality
logRelF0‐H1‐A3	Logarithmic ratio of F0 to harmonics, capturing voice roughness or breathiness
F1, F2, F3 frequency	Frequency of the formants (F1, F2, F3), related to vowel sound production
F1, F2, F3 bandwidth	Bandwidth of the formant (F1, F2, F3), indicative of vocal tract resonance
F1, F2, F3 amplitudeLogRelF0	Amplitude of formants (F1, F2, F3) relative to F0: vocal resonance and tonal quality

Features were extracted from the second‐by‐second automatically annotated acoustic signals. Separate time series were derived for the child and the caregiver, then aggregated at 500 ms. Prolonged moments without acoustic interactions (i.e., 25 s of complete silence from both speakers) were removed. When the corresponding speaker was silent, features from a placeholder silent audio segment were used. In case of concurrent vocalizations in the same second, features were extracted and assigned to both speaker vectors. Further, the feature time series of each speaker was independently standardized to ensure comparability.

#### 2.3.4 Cross‐Recurrence Quantification Analysis

CRQA is a non‐linear time‐series analysis technique based on complex dynamical systems theory. The aim of CRQA is to quantify the temporal coupling between two interacting systems, extending classical RQA by enabling the analysis of two separate time‐series. CRQA allows for the study of both synchronous and lagged interactions, producing a set of useful metrics that quantitatively describe recurring system properties (Marwan and Kurths 2002). Compared to more general techniques like cross‐correlation, CRQA can process non‐linear, non‐stationary, and complex relationships, including recursive ones, making it suitable for analyzing real‐world interactions and quantifying patterns of shared temporal structure and adaptation. In CRQA, two time series are embedded into a shared phase space, and a cross‐recurrence plot (CRP) is generated to identify all time points at which the states of the two systems are sufficiently similar based on Euclidean distance. The CRP is a binary matrix marking moments of shared or recurrent states between the two interacting signals. From this plot, CRQA metrics are derived. The recurrence rate (REC) measures the overall proportion of recurrent points, indicating how often the systems align. Determinism (DET) measures the proportion of recurrent points forming diagonal lines, reflecting predictable, sequential patterns in the interaction. Laminarity (LAM) measures the proportion of recurrent points forming vertical lines, reflecting periods where the systems remain in the same state. Composite metrics like the ratio between determinism and recurrence rate (DET/REC) indicate how much of the overall recurrence is structured and predictable rather than random, while the ratio between laminarity and determinism (LAM/DET) captures the balance between sustained stable states and sequential patterns. Together, these metrics describe both the frequency and the temporal organization of the dyad's coordination. Complementary measures further characterize the temporal organization of coordination. Average diagonal line length (*L*) indexes the typical duration of sequentially predictable states, while longest diagonal line (Lmax) highlights the longest sustained pattern of alignment. Trapping time (TT) measures the average length of vertical line structures, indicating how long systems remain in shared states. Entropy of diagonal line lengths (ENTR) quantifies the complexity and variability of sequential patterns, and entropy of vertical line lengths (V‐ENTR) does the same for laminar states. The CRQA metrics included in this work are summarized in Table 3.

**TABLE 3:** Summary of CRQA metrics (Marwan et al. 2007; Webber Jr and Zbilut 2005; Shockley 2005; Coco and Dale 2014).

Metric	Description	Interpretation	Increase	Decrease
Recurrence rate (REC)	Proportion of recurrent points in the recurrence plot	How often two time‐series revisit the same state	Increased synchronization or common influences	Weaker interaction or increased randomness in the system
Determinism (DET)	Proportion of recurrence points forming diagonal lines	Higher DET suggests more predictable and structured interactions	Stronger temporal dependence and shared dynamic patterns	More stochastic behavior or less structured coordination
Laminarity (LAM)	Proportion of recurrence points forming vertical lines	Reflects intermittency: higher LAM reflects tendency to remain in similar states	Increased stability or slowly gradual changes, but may also indicate rigid behavior or “stuck interactions”	More dynamic or less constrained interaction, perhaps also more unpredictable or unorganized
Determinism to recurrence rate ratio (DET/REC)	Ratio of DET to REC. Measures structure relative to recurrence	Higher values suggest more structured, less random recurrence patterns	Interactions are increasingly systematic and rule‐governed	Interactions are becoming more random or less structured
Laminarity to determinism ratio (LAM/DET)	Ratio of LAM to DET. Balances intermittency and predictability	Higher values indicate dominance of stability over predictable sequences	Prolonged stable states with fewer transitions with respect to overall predictability	Less persistent interactions, less stable states with respect to overall predictability

Abbreviations: DET, determinism; DET/REC, determinism to recurrence rate ratio; LAM, laminarity; LAM/DET, laminarity to determinism ratio; REC, recurrence rate.

### 2.4 Data Analysis Plan

Prosodic acoustic feature signals for both child and adult were robustly scaled using the median and interquartile range (IQR) to reduce the influence of outliers and ensure comparability across speakers and sessions.

CRQA was then performed using standard settings for vocal interaction data, with an embedding dimension of 3, a time delay of 3, and a fixed Euclidean radius of 1.5. This configuration balances sensitivity to fine‐grained temporal structure with robustness to noise and natural variability in spontaneous speech, allowing the method to capture short‐timescale vocal coordination patterns while maintaining sufficient temporal resolution to track adaptive fluctuations in interactional dynamics. Descriptive statistics were first calculated separately for mothers and fathers at both time points. We then conducted an exploratory correlation analysis to examine associations between synchrony indices and developmental and parental outcomes. To account for the large number of comparisons and to provide a more interpretable measure of evidential strength, Bayesian correlation tests were employed. Only associations with Bayes factors (BF) > 10, representing strong evidence for the alternative hypothesis, are reported. In case of violations of normality, rank‐based normalization was performed.

For data modeling, we employed a longitudinal, repeated‐measures design using linear mixed‐effects models. For each acoustic feature, we fitted two models with PSI total score, caregiver, and time as fixed effects, and subject as random intercept to model for interindividual variability. One model used the LAM/DET ratio as the dependent variable, while the second model was fitted with the DET/REC ratio of the corresponding acoustic feature as the dependent variable. Each model was tested against a baseline model without the fixed effect of stress to assess significance through the likelihood ratio test with chi‐squared (*X* ^2^) values, Akaike information criteria (AIC), and Bayesian information criteria (BIC) as complementary metrics.

Due to the reduced sample size, we performed a sensitivity analysis using a leave‐one‐subject‐out procedure with parametric bootstrap (*N* = 1000 simulations) to obtain an empirical *p*‐value for model significance together with average metrics for model terms. At each resampling step, the data were independently standardized. Models were checked for assumptions. In case of assumption violations, we used robust estimates of predictor significance based on bias‐reduced linearization adjustment (Bell and McCaffrey 2002; Pustejovsky and Tipton 2018). Fixed effect significance was used to evaluate the impact of parental stress, caregiver, and changes over time.

**FIGURE 1:**
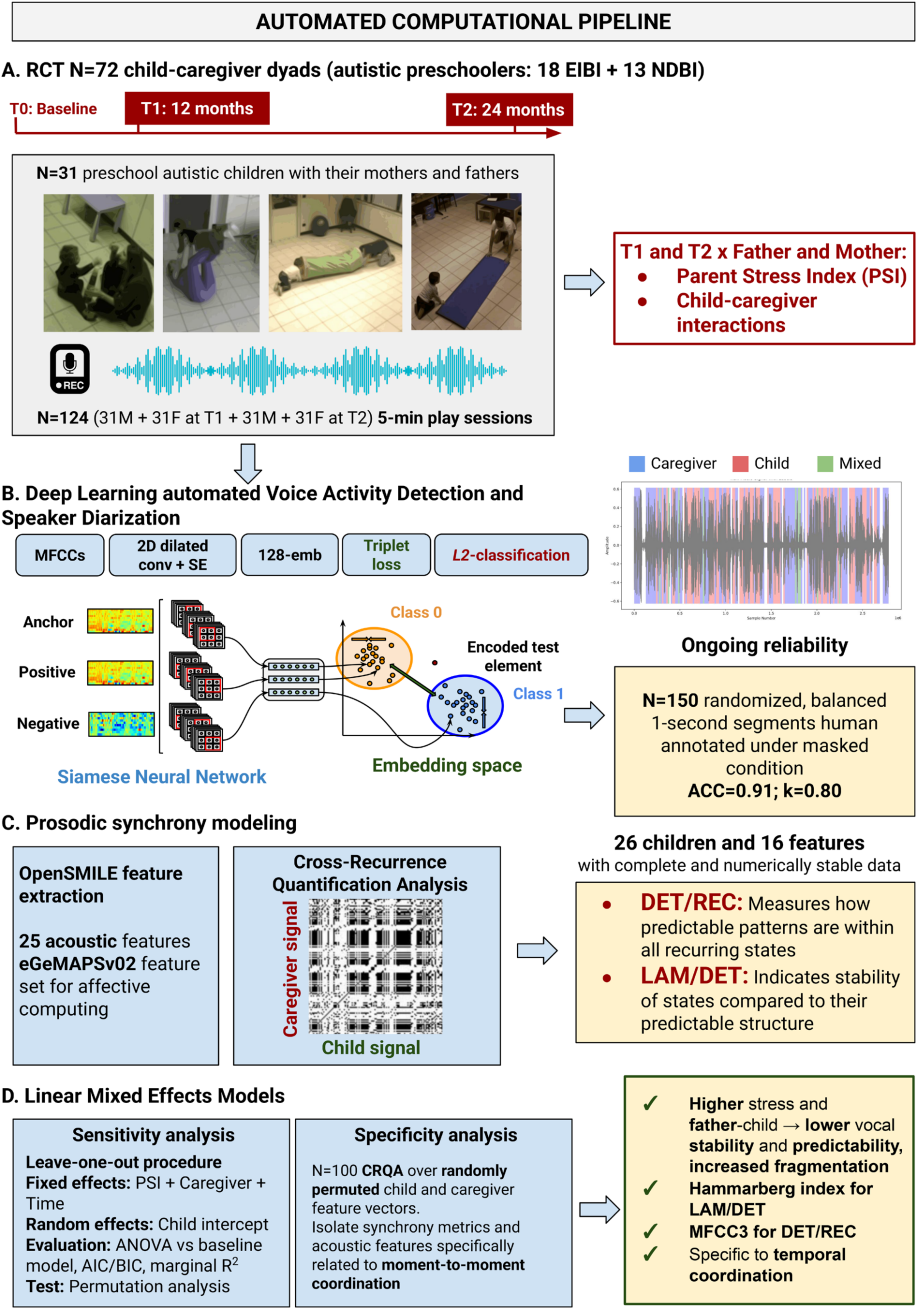
AI‐based, automated computational pipeline leveraging complex systems theory and affective computing.

The second step of the data analysis plan (DAP) involved specificity analysis to investigate further acoustic features related to moment‐to‐moment coordination or general prosodic coupling. We expected that child–caregiver prosodic interaction may exhibit synchronization at different levels. For our study, we were particularly interested in understanding whether acoustic synchronization relates to general aspects (i.e., not strictly time‐dependent) or specifically to moment‐to‐moment coordination in the acoustic interaction. To achieve this, we generated a null distribution by permuting feature vectors while preserving individual speech characteristics but disrupting dyadic temporal alignment (*N* = 100). This allowed computation of empirically‐based specificity *p*‐values for model significance and model terms based on chi‐squared and *t* statistic distributions. Models were prioritized sequentially: first those showing overall significance from permutation testing, then those demonstrating specificity to true temporal coordination from the randomization analysis, further those with a significant parenting stress effect from internal sensitivity testing, and finally those also evidencing specificity of the stress effect to temporal coordination. Multiple comparisons were controlled by Monte Carlo family‐wise Type I error simulation (*N* = 50,000 iterations). Models specific to temporal coordination were retained, and only models that also showed a temporal‐dynamic specific effect of stress passed the final selection. Selected models were evaluated using marginal *R* ^2^ and fixed effects. Monte Carlo simulation showed that the probability of at least one false positive surviving the first two selection steps was 0.08, whereas the probability of passing the final selection was approximately 0.0003. Simulation‐based power analysis (*N* = 5000) indicated an approximate power of 0.74 (95% CI = [0.73, 0.75]) for detecting a medium effect size for PSI (*b* = 0.25), an approximate power of 0.78 (95% CI = [0.77, 0.79]) for detecting a medium effect size for caregiver role (*b* = 0.35), and an approximate power of 0.75 (95% CI = [0.74, 0.76]) for detecting a medium effect size of time (*b* = 0.35). For our model comparison, estimated power was 0.72 (95% CI = [0.71, 0.73]). The design of this study including the full analytic pipeline is schematized in Figure 1.

## 3 Results

### 3.1 Cross‐Recurrence Quantification Analysis

CRQA showed moderate levels of system recurrence (REC) together with moderate‐to‐high presence of deterministic structures in the signal (DET). The analysis highlighted a significant presence of stable acoustic states characterizing the acoustic interaction, indicating that acoustic features tended to remain in particular states for prolonged periods of sustained vocalizations or consistent acoustic patterns (LAM). CRQA indicated a slight dominance of structured patterns over the total recurrence, indicating predictability (DET/REC). As well, the stability of acoustic states was slightly higher than their overall predictability, indicating sustained vocalizations within the structured interaction. Data were pre‐processed, ensuring the full availability of clinical data and acoustic interactions for both caregivers at both time points. We also excluded features that showed numerical instability in the CRQA analysis and retained only features with complete data for all the children. After this process, 26 children were retained for the synchrony analysis, together with 16 features. Therefore, the threshold for Bonferroni correction was set to *p* = 0.002, as significance tests were performed over all 32 models. Summary statistics of synchrony metrics are reported in Table 4. At T1, Bayesian correlation analyses showed a negative association between the Vineland Composite Score (VABS‐II—CS) and DET for logRelF0‐H1‐H2 (*r* = −0.53; BF = 12), observed only in mother–child dyads. At T2, DET for MFCC2 was positively associated with PSI Total Score in father–child dyads (*r* = 0.59; BF = 27). In fathers, higher PSI Total score was also associated with lower LAM/DET values for F1 amplitude (*r* = −0.62; BF = 48), F2 amplitude (*r* = −0.62; BF = 46), and F3 amplitude (*r* = −0.61; BF = 40), and with higher REC values for MFCC1 (*r* = 0.52; BF = 10). Finally, both REC (*r* = −0.55; BF = 14) and DET (*r* = −0.62; BF = 52) for F1 frequency were negatively associated with the GMDS‐ER general developmental quotient (GMDS‐ER GDQ), again specifically in father–child dyads.

**TABLE 4:** Summary statistics of synchrony metrics.

Feature	Role	T1					T2				
		REC	DET	LAM	DET/REC	LAM/DET	REC	DET	LAM	DET/REC	LAM/DET
F0semitoneFrom27.5 Hz	Father	0.48 (0.10)	0.80 (0.07)	0.86 (0.05)	1.72 (0.25)	1.08 (0.04)	0.45 (0.09)	0.78 (0.08)	0.85 (0.05)	1.77 (0.19)	1.09 (0.06)
F0semitoneFrom27.5 Hz	Mother	0.39 (0.08)	0.74 (0.08)	0.83 (0.06)	1.95 (0.28)	1.12 (0.06)	0.39 (0.09)	0.76 (0.08)	0.84 (0.06)	1.98 (0.27)	1.11 (0.05)
F1amplitudeLogRelF0	Father	0.28 (0.07)	0.61 (0.11)	0.70 (0.11)	2.18 (0.20)	1.16 (0.07)	0.26 (0.05)	0.58 (0.08)	0.69 (0.10)	2.28 (0.25)	1.18 (0.09)
F1amplitudeLogRelF0	Mother	0.27 (0.04)	0.59 (0.07)	0.71 (0.09)	2.19 (0.16)	1.20 (0.06)	0.28 (0.03)	0.61 (0.05)	0.73 (0.07)	2.17 (0.15)	1.20 (0.06)
F1frequency	Father	0.17 (0.10)	0.53 (0.20)	0.61 (0.21)	3.75 (1.46)	1.17 (0.09)	0.19 (0.10)	0.58 (0.17)	0.69 (0.18)	3.45 (1.04)	1.21 (0.12)
F1frequency	Mother	0.14 (0.06)	0.47 (0.16)	0.62 (0.21)	3.57 (0.92)	1.37 (0.23)	0.14 (0.06)	0.50 (0.16)	0.65 (0.19)	4.03 (1.56)	1.30 (0.17)
F2amplitudeLogRelF0	Father	0.28 (0.07)	0.61 (0.11)	0.70 (0.11)	2.22 (0.21)	1.16 (0.07)	0.25 (0.05)	0.58 (0.08)	0.69 (0.10)	2.33 (0.26)	1.19 (0.09)
F2amplitudeLogRelF0	Mother	0.27 (0.04)	0.59 (0.07)	0.71 (0.09)	2.23 (0.16)	1.21 (0.07)	0.27 (0.04)	0.60 (0.06)	0.72 (0.08)	2.23 (0.16)	1.20 (0.06)
F3amplitudeLogRelF0	Father	0.27 (0.06)	0.61 (0.11)	0.70 (0.11)	2.25 (0.21)	1.16 (0.07)	0.25 (0.05)	0.58 (0.07)	0.69 (0.10)	2.35 (0.26)	1.19 (0.09)
F3amplitudeLogRelF0	Mother	0.26 (0.04)	0.58 (0.07)	0.70 (0.10)	2.25 (0.16)	1.21 (0.07)	0.26 (0.03)	0.59 (0.06)	0.72 (0.08)	2.25 (0.17)	1.21 (0.06)
HNRdBACF	Father	0.57 (0.09)	0.85 (0.06)	0.90 (0.04)	1.52 (0.15)	1.06 (0.03)	0.55 (0.08)	0.84 (0.06)	0.90 (0.03)	1.55 (0.13)	1.07 (0.05)
HNRdBACF	Mother	0.48 (0.08)	0.80 (0.05)	0.88 (0.04)	1.69 (0.18)	1.09 (0.04)	0.48 (0.08)	0.80 (0.06)	0.87 (0.04)	1.71 (0.20)	1.09 (0.04)
Loudness	Father	0.51 (0.12)	0.83 (0.08)	0.88 (0.06)	1.69 (0.28)	1.06 (0.04)	0.48 (0.13)	0.82 (0.08)	0.88 (0.05)	1.78 (0.28)	1.08 (0.06)
Loudness	Mother	0.45 (0.13)	0.79 (0.09)	0.86 (0.06)	1.86 (0.35)	1.10 (0.05)	0.45 (0.10)	0.80 (0.07)	0.87 (0.06)	1.85 (0.28)	1.09 (0.04)
alphaRatio	Father	0.38 (0.07)	0.72 (0.09)	0.80 (0.08)	1.95 (0.17)	1.11 (0.05)	0.35 (0.06)	0.70 (0.07)	0.80 (0.06)	2.05 (0.20)	1.14 (0.08)
alphaRatio	Mother	0.34 (0.04)	0.69 (0.04)	0.80 (0.04)	2.06 (0.16)	1.15 (0.05)	0.34 (0.05)	0.70 (0.05)	0.81 (0.04)	2.09 (0.16)	1.15 (0.06)
hammarbergIndex	Father	0.29 (0.08)	0.67 (0.09)	0.75 (0.08)	2.40 (0.34)	1.14 (0.06)	0.26 (0.06)	0.65 (0.09)	0.75 (0.08)	2.54 (0.29)	1.17 (0.10)
hammarbergIndex	Mother	0.24 (0.03)	0.62 (0.06)	0.75 (0.08)	2.58 (0.20)	1.21 (0.07)	0.24 (0.05)	0.63 (0.07)	0.75 (0.07)	2.62 (0.29)	1.20 (0.08)
logRelF0‐H1‐A3	Father	0.52 (0.08)	0.82 (0.05)	0.88 (0.04)	1.62 (0.17)	1.07 (0.03)	0.49 (0.08)	0.80 (0.06)	0.86 (0.04)	1.67 (0.16)	1.08 (0.05)
logRelF0‐H1‐A3	Mother	0.45 (0.07)	0.77 (0.06)	0.85 (0.04)	1.76 (0.19)	1.10 (0.05)	0.45 (0.09)	0.78 (0.07)	0.86 (0.05)	1.76 (0.19)	1.10 (0.04)
logRelF0‐H1‐H2	Father	0.59 (0.09)	0.85 (0.06)	0.91 (0.04)	1.47 (0.12)	1.07 (0.03)	0.56 (0.07)	0.84 (0.05)	0.89 (0.03)	1.51 (0.10)	1.07 (0.04)
logRelF0‐H1‐H2	Mother	0.51 (0.07)	0.81 (0.06)	0.88 (0.05)	1.60 (0.12)	1.09 (0.03)	0.52 (0.07)	0.82 (0.05)	0.89 (0.04)	1.59 (0.13)	1.08 (0.03)
mfcc1	Father	0.35 (0.08)	0.72 (0.08)	0.80 (0.07)	2.11 (0.25)	1.12 (0.05)	0.31 (0.05)	0.68 (0.07)	0.78 (0.07)	2.22 (0.20)	1.15 (0.08)
mfcc1	Mother	0.29 (0.04)	0.66 (0.05)	0.77 (0.06)	2.27 (0.19)	1.18 (0.06)	0.30 (0.05)	0.68 (0.06)	0.79 (0.04)	2.31 (0.31)	1.17 (0.07)
mfcc2	Father	0.43 (0.07)	0.75 (0.06)	0.82 (0.05)	1.77 (0.15)	1.09 (0.03)	0.42 (0.06)	0.76 (0.06)	0.83 (0.05)	1.80 (0.15)	1.11 (0.05)
mfcc2	Mother	0.40 (0.04)	0.74 (0.04)	0.82 (0.04)	1.85 (0.11)	1.12 (0.04)	0.41 (0.04)	0.75 (0.04)	0.83 (0.04)	1.86 (0.10)	1.11 (0.03)
mfcc3	Father	0.46 (0.07)	0.79 (0.05)	0.85 (0.04)	1.73 (0.15)	1.07 (0.03)	0.44 (0.07)	0.77 (0.06)	0.83 (0.05)	1.76 (0.15)	1.09 (0.04)
mfcc3	Mother	0.41 (0.06)	0.74 (0.05)	0.82 (0.04)	1.84 (0.16)	1.11 (0.04)	0.41 (0.06)	0.75 (0.06)	0.83 (0.05)	1.83 (0.17)	1.11 (0.04)
mfcc4	Father	0.47 (0.05)	0.78 (0.05)	0.85 (0.04)	1.69 (0.12)	1.08 (0.04)	0.44 (0.06)	0.77 (0.05)	0.84 (0.04)	1.79 (0.16)	1.10 (0.05)
mfcc4	Mother	0.41 (0.05)	0.75 (0.04)	0.84 (0.03)	1.82 (0.16)	1.12 (0.04)	0.42 (0.05)	0.76 (0.04)	0.84 (0.03)	1.84 (0.14)	1.11 (0.03)
shimmerLocaldB	Father	0.49 (0.09)	0.80 (0.07)	0.86 (0.05)	1.67 (0.19)	1.08 (0.04)	0.45 (0.08)	0.77 (0.08)	0.84 (0.06)	1.72 (0.17)	1.09 (0.05)
shimmerLocaldB	Mother	0.41 (0.07)	0.74 (0.06)	0.83 (0.05)	1.83 (0.20)	1.12 (0.05)	0.41 (0.08)	0.75 (0.08)	0.83 (0.06)	1.86 (0.20)	1.11 (0.05)

### 3.2 Linear Mixed Models With and Without Parental Stress

A total of 10 models were found to be significant with respect to comparisons with baseline models without the stress term: two for the determinism to recurrence rate (DET/REC) ratio and eight for the laminarity to determinism ratio (LAM/DET). For LAM/DET, the stepwise selection process retained five models for the first two steps for general specificity, whereas one model passed the whole selection. LAM/DET describes the balance between stability and predictability and was associated with four acoustic features (F1, F2, and F3 amplitudes, and Hammarberg index). Hammarberg index was specifically associated with parental stress and to temporal coordination, which negatively impacted LAM/DET, meaning that higher levels of stress were associated with interactions that had a lower LAM/DET. The child–father interaction (as opposed to the child–mother interaction) was also characterized by a significantly lower LAM/DET. No effects of time emerged. The caregiver effect was specific to temporal coordination. In summary, fathers and more subjectively stressed caregivers exhibited a prosodic interaction characterized by less stability and higher fluctuations. On the contrary, mothers and less stressed caregivers exhibited prolonged stable interactions with slower, more gradual changes. F1, F2, and F3 amplitudes are acoustic markers that reflect the resonant properties of the vocal tract and are closely related to articulation and voice quality. The Hammarberg Index measures the spectral tilt of the voice by comparing the energy between low and high frequency bands. Fixed effects (PSI: *b* = −0.19, SE = 0.07; caregiver *b* = −0.42, SE = 0.14; time *b* = 0.09, SE = 0.09) of the final selected model (Hammarberg index) explained 17% of the observed variance (compared to 10% in the baseline model).

The stepwise selection process retained two models for DET/REC after the first two steps, with one model selected by the whole procedure. Concerning the determinism to recurrence rate ratio (DET/REC), which measures the balance between recurrent states and system predictability (i.e., the presence of a deterministic structure and not just repetition of similar states), the third MFCC was negatively associated with parental stress and DET/REC, meaning that higher levels of stress implied interactions with a lower DET/REC. This association was specific to child‐caregiver temporal alignment. Additionally, fathers showed prosodic interactions with a significantly lower DET/REC. The effect of caregiver was not specific to temporal coordination. In summary, fathers and more stressed caregivers showed acoustic interactions characterized by a lower degree of internal structure and increased randomness. On the contrary, mothers and less stressed caregivers exhibited a prosodic interaction characterized by a significantly greater internal, deterministic, and predictable structure in terms of recurrence profile. Fixed effects (PSI: *b* = −0.20, SE = 0.08; caregiver *b* = −0.34, SE = 0.12; time *b* = 0.10, SE = 0.09) of the final selected model (third MFCC) explained 17% of the observed variance (compared to 8% in the baseline model).

Tables 5 and 6 report the full results. The summary of the complete analytic pipeline can be found in the Appendix S1.

**TABLE 5:** Linear mixed effect models over the determinism to recurrence rate (DET/REC) ratio.

Feature	Determinism/recurrence rate (DET/REC)										
	*X* ^2^	*p*‐ANOVA	*p*‐dist	*p*‐rand	*p*‐rand‐dist	*p*‐stress	*p*‐stress‐dist	*p*‐caregiver	*p*‐caregiver‐dist	*p*‐time	*p*‐time‐dist
alphaRatio	3.300	0.083	> 0.05	0.305	> 0.05	0.130	> 0.05	0.049	> 0.05	0.038	> 0.05
F0semitoneFrom27.5 Hz	1.755	0.197	> 0.05	0.341	> 0.05	0.179	> 0.05	0.000	< 0.001	0.221	> 0.05
F1amplitudeLogRelF0	0.134	0.761	> 0.05	0.573	> 0.05	0.801	> 0.05	0.300	> 0.05	0.186	> 0.05
F1frequency	0.081	0.826	> 0.05	0.412	> 0.05	0.840	> 0.05	0.447	> 0.05	0.734	> 0.05
F2amplitudeLogRelF0	0.116	0.779	> 0.05	0.562	> 0.05	0.818	> 0.05	0.347	> 0.05	0.093	> 0.05
F3amplitudeLogRelF0	0.158	0.735	> 0.05	0.520	> 0.05	0.780	> 0.05	0.327	> 0.05	0.124	> 0.05
hammarbergIndex	0.224	0.676	> 0.05	0.444	> 0.05	0.689	> 0.05	0.015	< 0.001	0.077	> 0.05
HNRdBACF	1.811	0.192	> 0.05	0.317	> 0.05	0.213	> 0.05	0.000	< 0.001	0.206	> 0.05
logRelF0‐H1‐A3	1.642	0.216	> 0.05	0.219	> 0.05	0.268	> 0.05	0.001	< 0.001	0.252	> 0.05
logRelF0‐H1‐H2	1.699	0.201	> 0.05	0.176	> 0.05	0.167	> 0.05	0.000	< 0.001	0.294	> 0.05
Loudness	1.905	0.180	> 0.05	0.321	> 0.05	0.185	> 0.05	0.008	< 0.001	0.418	> 0.05
mfcc1	0.321	0.609	> 0.05	0.474	> 0.05	0.639	> 0.05	0.013	< 0.001	0.196	> 0.05
mfcc2	3.879	0.056	> 0.05	0.293	> 0.05	0.063	> 0.05	0.008	< 0.001	0.340	> 0.05
**mfcc3**	**7.034**	**0.010**	**< 0.001**	**0.142**	**> 0.05**	**0.011**	**< 0.001**	**0.001**	**< 0.001**	**0.389**	**> 0.05**
mfcc4	0.552	0.493	> 0.05	0.300	> 0.05	0.562	> 0.05	0.001	< 0.001	0.003	< 0.001
shimmerLocaldB	3.287	0.077	> 0.05	0.267	> 0.05	0.097	> 0.05	0.000	< 0.001	0.091	> 0.05

*Note*: Significant models which proved to be specifically related to moment‐to‐moment temporal coordination are marked in bold.

Abbreviations: *p*‐ANOVA, the average *p*‐value for the ANOVAs of the sensitivity analysis; *p*‐caregiver, the average *p*‐value for the caregiver term of the linear mixed model; *p*‐caregiver‐dist, the *p*‐value for the null hypothesis that the average *p*‐value of the sensitivity analysis was lower than 0.05 for the caregiver term; *p*‐dist, the *p*‐value for the null hypothesis that the average *p*‐value of the sensitivity analysis was lower than 0.05 for the ANOVA; *p*‐rand, the average *p*‐value for the ANOVAs of the permuted sensitivity analysis; *p*‐rand‐dist, the *p*‐value for the null hypothesis that the average *p*‐value of the permuted sensitivity analysis was lower than 0.05 for the ANOVA; *p*‐stress, the average *p*‐value for the stress term of the linear mixed model; *p*‐stress‐dist, the *p*‐value for the null hypothesis that the average *p*‐value of the sensitivity analysis was lower than 0.05 for the stress term; *p*‐time, the average *p*‐value for the time term of the linear mixed model; *p*‐time‐dist, the *p*‐value for the null hypothesis that the average *p*‐value of the sensitivity analysis was lower than 0.05 for the time term; *X* ^2^, the average chi‐squared value for the ANOVAs of the sensitivity analysis.

**TABLE 6:** Linear mixed effect models over the laminarity to determinism (LAM/DET) ratio.

Feature	Laminarity/determinism (LAM/DET)										
	*X* ^2^	*p*‐ANOVA	*p*‐dist	*p*‐rand	*p*‐rand‐dist	*p*‐stress	*p*‐stress‐dist	*p*‐caregiver	*p*‐caregiver‐dist	*p*‐time	*p*‐time‐dist
alphaRatio	2.897	0.107	> 0.05	0.028	< 0.001	0.198	> 0.05	0.048	> 0.05	0.050	> 0.05
F0semitoneFrom27.5 Hz	2.036	0.168	> 0.05	0.047	> 0.05	0.212	> 0.05	0.007	< 0.001	0.874	> 0.05
**F1amplitudeLogRelF0**	**7.197**	**0.010**	**< 0.001**	**0.259**	**> 0.05**	**0.011**	**< 0.001**	**0.031**	**< 0.001**	**0.230**	**> 0.05**
F1frequency	1.332	0.271	> 0.05	0.615	> 0.05	0.327	> 0.05	0.001	< 0.001	0.637	> 0.05
**F2amplitudeLogRelF0**	**7.365**	**0.010**	**< 0.001**	**0.328**	**> 0.05**	**0.026**	**< 0.001**	**0.053**	**> 0.05**	**0.189**	**> 0.05**
**F3amplitudeLogRelF0**	**7.501**	**0.009**	**< 0.001**	**0.395**	**> 0.05**	**0.009**	**< 0.001**	**0.022**	**< 0.001**	**0.163**	**> 0.05**
**hammarbergIndex**	**5.154**	**0.027**	**< 0.001**	**0.626**	**> 0.05**	**0.028**	**< 0.001**	**0.007**	**< 0.001**	**0.169**	**> 0.05**
HNRdBACF	1.852	0.191	> 0.05	0.085	> 0.05	0.245	> 0.05	0.002	< 0.001	0.679	> 0.05
logRelF0‐H1‐A3	3.169	0.083	> 0.05	0.025	< 0.001	0.106	> 0.05	0.014	< 0.001	0.261	> 0.05
logRelF0‐H1‐H2	4.784	0.036	< 0.001	0.012	< 0.001	0.087	> 0.05	0.049	> 0.05	0.433	> 0.05
Loudness	1.721	0.203	> 0.05	0.027	< 0.001	0.204	> 0.05	0.019	< 0.001	0.737	> 0.05
mfcc1	4.034	0.053	> 0.05	0.254	> 0.05	0.089	> 0.05	0.011	< 0.001	0.068	> 0.05
mfcc2	1.811	0.197	> 0.05	0.022	< 0.001	0.218	> 0.05	0.114	> 0.05	0.712	> 0.05
mfcc3	5.721	0.019	< 0.001	0.021	< 0.001	0.023	< 0.001	0.000	< 0.001	0.156	> 0.05
mfcc4	4.484	0.043	> 0.05	0.083	> 0.05	0.081	> 0.05	0.018	< 0.001	0.519	> 0.05
shimmerLocaldB	4.136	0.048	> 0.05	0.034	< 0.001	0.063	> 0.05	0.004	< 0.001	0.429	> 0.05

*Note*: Significant models which proved to be specifically related to moment‐to‐moment temporal coordination are marked in bold.

Abbreviations: *p*‐ANOVA, the average *p*‐value for the ANOVAs of the sensitivity analysis; *p*‐caregiver, the average *p*‐value for the caregiver term of the linear mixed model; *p*‐caregiver‐dist, the *p*‐value for the null hypothesis that the average *p*‐value of the sensitivity analysis was lower than 0.05 for the caregiver term; *p*‐dist, the *p*‐value for the null hypothesis that the average *p*‐value of the sensitivity analysis was lower than 0.05 for the ANOVA; *p*‐rand, the average *p*‐value for the ANOVAs of the permuted sensitivity analysis; *p*‐rand‐dist, the *p*‐value for the null hypothesis that the average *p*‐value of the permuted sensitivity analysis was lower than 0.05 for the ANOVA; *p*‐stress, the average *p*‐value for the stress term of the linear mixed model; *p*‐stress‐dist, the *p*‐value for the null hypothesis that the average *p*‐value of the sensitivity analysis was lower than 0.05 for the stress term; *p*‐time, the average *p*‐value for the time term of the linear mixed model; *p*‐time‐dist, the *p*‐value for the null hypothesis that the average *p*‐value of the sensitivity analysis was lower than 0.05 for the time term; *X* ^2^, the average chi‐squared value for the ANOVAs of the sensitivity analysis.

## 4 Discussion

The present study investigated the role of parental stress, caregiver role differences, and longitudinal evolution in shaping the temporal dynamics of acoustic synchrony during naturalistic child‐caregiver interactions. Using CRQA and a permutation‐based analytic strategy grounded in dynamic systems theory and affective computing, we explored how parental stress and caregiver role modulated the moment‐to‐moment coordination of acoustic features.

Correlation patterns provide preliminary insight into how caregiver stress and child developmental level may relate to fine‐grained vocal coordination dynamics within the dyad. At baseline, higher child adaptive functioning was associated with lower sequential predictability (DET) in maternal prosodic alignment, suggesting that for mothers, children with stronger adaptive profiles may engage in interactions characterized by more flexible and less tightly patterned vocal coordination. Across time, the stronger associations emerged primarily in father–child dyads. Higher paternal stress was linked to greater predictability in MFCC‐based coordination and reduced stability in sustained patterns of amplitude alignment across formant frequencies, alongside increased general recurrence in the first MFCC. These patterns may reflect a shift toward more regular and less varied vocal coupling in the context of elevated paternal stress. Furthermore, both recurrence and determinism in F1 frequency coordination negatively correlated with child developmental level, indicating that lower child developmental functioning was associated with more tightly structured father–child coordination dynamics.

Our LMMs indicate that parental stress and caregiver role are associated with measurable differences in the temporal structure of child–caregiver prosodic coordination. Higher stress levels were associated with lower LAM/DET in spectral slope and lower DET/REC in the third MFCC, suggesting that more stressed caregivers engage in vocal exchanges with reduced stability in their moment‐to‐moment coupling and weaker deterministic structure. In recurrence‐analysis terms, these dyads spent less time in sustained shared vocal states and showed fewer orderly transitions in their acoustic alignment, reflecting a pattern of more fragmented and less temporally organized vocal coordination. Conversely, lower stress was associated with more recurrent, predictable, and stable vocal patterns, indicating smoother and more coherent temporal organization of the interaction. Caregiver role further modulated these dynamics, with specificity to moment‐to‐moment coordination for LAM/DET and Hammarberg index, where father–child dyads showed lower LAM/DET values compared to mother–child dyads. This implies that vocal turn‐taking with fathers tended to be characterized by faster shifts and less sustained shared acoustic states. By contrast, mothers exhibited prosodic dynamics marked by greater stability, spending longer intervals in aligned acoustic configurations and transitioning through recurrence patterns in a more orderly manner. For the third MFCC, mothers also showed a stronger deterministic structure in prosodic coupling, though this effect was not specifically associated with temporal‐coordination. These recurrence‐structure differences emerged without significant time effects, suggesting that the observed interaction styles may be role‐specific. Interestingly, while DET/REC reflects the proportion of deterministic versus random coupling, LAM/DET can decrease even when determinism increases, reflecting a system that is more dynamic and flexible rather than disorganized, with shorter periods of sustained states relative to overall sequential structure, potentially reflecting different interaction style sensible to stress. Importantly, our findings suggest that parental stress and caregiver role contribute to acoustic synchrony through partially distinct mechanisms. Stress‐related effects are more directly associated with moment‐to‐moment temporal coordination. In contrast, caregiver role differences appear to reflect more global interactional styles, especially for pattern predictability, which is less dependent on immediate temporal alignment and more indicative of broader prosodic organization across the interaction. This distinction suggests that while stress modulates how coordination unfolds from moment to moment, caregiver role may be linked to the overall structure within which these temporal dynamics occur.

The acoustic features involved perceptual and spectral aspects of speech, including formant amplitudes and spectral tilt, which have been implicated in emotional expression (Ekberg et al. 2023; Tamarit et al. 2008), and have also been shown to be related to stress (Sluijter and Van Heuven 1996; Yao et al. 2021). Variations in formant amplitudes can also signal differences in speech clarity, emotional expressiveness, or stress‐related vocal modulation (Iqbal et al. 2020; Madanian et al. 2023). Finally, the Hammarberg index is often interpreted as an indicator of vocal tension or effort, with higher values typically associated with strained or pressed phonation and lower values reflecting softer, more relaxed vocal quality. Together, these features are informative for assessing affective tone, vocal expressivity, and stress‐related changes in speech (Patel et al. 2011). Our findings seem consistent with prior research indicating that parenting stress is associated with altered affective communication and lower quality parent–child interactions (Azhari et al. 2019, 2022). Importantly, our analytic approach allows us to quantify these effects using interpretable metrics of dynamic coupling, capturing not only the presence but also the structure of interpersonal synchrony.

Clinical literature linked parental stress to reduced sensitivity, increased intrusiveness, and lower patience in caregiving (Dolev et al. 2009; Zaidman‐Zait et al. 2014). The unique communication and behavioral styles of children with ASD can further challenge parental responsiveness and confidence, potentially weakening the parent–child interaction (Van IJzendoorn et al. 2007).

Our data showed that parental stress mainly impacts the stability of interactional patterns between caregivers and children with autism. Higher levels of stress are associated with interaction patterns that are less sustained, more dynamic or chaotic. Stressed parents may feel overwhelmed and unable to cope with the situation, or they may perceive that their child is not enjoying the interaction, therefore trying out various strategies in an attempt to engage them (Davis and Carter 2008; Hoffman et al. 2009; Kasari and Sigman 1997). Moreover, as is often the case in autism, when the child does not initiate or seek interaction, the parent may experience increased stress and respond by frequently altering their interactive approach (Giallo et al. 2013; Valeri et al. 2020). Indeed, fathers and more stressed caregivers seem to continuously adjust their behavior, resulting in less predictable and sustained interactions (Bradley et al. 2024). Parental stress mainly impacts the stability of interactional patterns between caregivers and autistic children. Higher levels of stress were associated with interaction patterns that are less sustained and more dynamically fluctuating. Rather than reflecting child‐driven interactional difficulties, these patterns could reflect differences in how caregivers implement behavioral adjustments under stress. Stressed caregivers may engage in frequent attempts to adapt their vocal and interactional strategies. This is particularly relevant for autistic children, where the ability to follow the child's lead and co‐construct predictable and stable even if varied, interactional routines is strongly associated with positive developmental outcomes, both cognitive and socio‐emotional (Cheng et al. 2023; Hajal and Paley 2020; McDowell and Parke 2009; Swanson et al. 2011).

Our findings also emphasize the potential of acoustic features as low‐level, objective indicators of relational quality in early interactions. They suggest that affective cues embedded in the prosodic signal may be used not only to infer caregiver states but also to characterize the dynamic flow of interactional exchanges.

Father‐child acoustic interactions exhibited less stable states, less smooth transitions, and lower deterministic structure in spectral aspects. This may be due to the fact that fathers often engage in more horizontal interactions, which can encourage mutual exploration and spontaneous communication in autistic children resulting in more varied interaction patterns (Bornstein and Venuti 2013; Elder et al. 2003; Flippin and Watson 2015). While this approach can result in less predictable synchrony, it may also reflect a valuable dimension of the interaction (Bentenuto et al. 2020; Flippin and Crais 2011; Siller and Sigman 2008). Fathers may also interact less frequently with their children on a daily basis, potentially displaying greater difficulty in sustaining the interaction (Oppenheim et al. 2025). It is important to emphasize that these different types of synchrony may complement each other, both serving adaptive roles for child development. While previous research has documented differences in maternal and paternal interactional styles (Azhari et al. 2022; Cohen et al. 2013; Feldman 2003; Paquette 2004), our results provide fine‐grained temporal evidence of how these differences manifest in the acoustic domain. For example, developmental research indicates that, while interacting with their children, mothers tend to prioritize structuring prolonged attunement more oriented toward teaching and vertical aspects whereas fathers exhibit a more playful, horizontal style in a complementary manner (Feldman 2003; Paquette 2004). It is also possible that fathers and mothers exhibit a diverse set of strengths and weaknesses, which should be carefully considered. It is essential to monitor and address parental stress to enhance therapeutic interventions and promote the well‐being of the entire family (Cheng et al. 2023; Edmunds and Hock 2025).

A key strength of our analytic framework lies in its ability to isolate the temporal dependencies underlying acoustic synchrony. The use of a permutation‐based strategy provided critical validation for the specificity of our findings. By comparing observed synchrony metrics with null distributions generated from temporally decoupled data, we showed that the associations we observed were not driven by general properties of the acoustic signal but by genuine moment‐to‐moment coordination between caregiver and child, in line with the construct of synchrony (Bernieri et al. 1988; Delaherche et al. 2012).

This study is not without limitations, the main one being the reduced sample size, which, despite our rigorous analysis, demands cautious interpretation of results and limits their generalizability, and also increases Type I and Type II error rates. Replication in larger, more diverse cohorts is necessary to confirm the robustness of these patterns and explore potential moderating factors, associations with clinical outcomes, and features linked to autism in relation to typical development. Additionally, despite the longitudinal design, this study is inherently correlational. Therefore, causal or predictive pathways cannot be established. Further research should explore these aspects, as well as multimodal features related to the child–caregiver interplay and communication. Future research should aim to delineate synchrony profiles linked to developmental and behavioral outcomes, in order to specifically map adaptive and maladaptive interaction patterns.

To conclude, our findings highlight key acoustic markers associated with parental stress, provide additional empirical support for the use of recurrence‐based approaches in the analysis of dyadic synchrony, and offer novel insights into the differential interactional patterns exhibited by mothers and fathers with autistic children. Differences in prosodic synchrony specific to caregiver roles may also reflect a mechanism of caregiver adaptation to the individual characteristics of the child.

## Funding

This work was supported by the Italian Ministry of Health with “Current Research funds”.

## Ethics Statement

This study was conducted according to guidelines laid down in the Declaration of Helsinki, with written informed consent obtained from a parent for each infant before any assessment or data collection. The sample was treated in accordance with the ethical standards outlined by the American Psychological Association and the Italian Association of Academic Psychologists. This study was reviewed and approved by the Ethical Committee of the Istituto Superiore di Sanità (Rome, Italy), which approved all components of the experimental protocol and methods described in this paper (code: WFR‐NET‐2013‐02355263).

## Consent

Written informed consent was obtained from all the patients included in the study.

## Conflicts of Interest

The authors declare no conflicts of interest.

## Supporting information


**APPENDIX S1:** Supporting information.

## Data Availability

The data that support the findings of this study are available on request from the corresponding author. The data are not publicly available due to privacy or ethical restrictions.
